# Study on the Influence of Graphene Content Variation on the Microstructure Evolution and Properties of Laser Additive Manufacturing Nickel-Based/SiC Composite Cladding Layer on Aluminum Alloy Surface

**DOI:** 10.3390/ma15228219

**Published:** 2022-11-18

**Authors:** Fuzhen Sun, Xiaoxu Li, Kaiyuan Zheng, Bo Han, Yan Li, Yong Zang, Ming Pang

**Affiliations:** 1 State Key Laboratory of Advanced Forming Technology and Equipment, China Academy of Machinery Science & Technology, Beijing 100044, China; 2 School of Mechanical Engineering, University of Science & Technology Beijing, Beijing 100083, China; 3 College of Aeronautical Engineering, Civil Aviation University of China, Tianjin 300300, China

**Keywords:** laser additive, Ni-based superalloy layer, wear resistance, graphene content

## Abstract

A Ni25—10% SiC—X% graphene (mass fraction X = 0, 0.5, 1.0, 1.5) composite cladding layer was prepared on a 6063 aluminum alloy substrate using laser cladding in order to enhance the comprehensive performance of the aircraft refueling interface. The effect of the graphene content on the organization and properties of nickel-based silicon carbide composite cladding layers was investigated by laser melting. The macroscopic morphology, microstructure, hardness, elemental changes, corrosion and wear resistance of the cladding layer were studied by optical microscopy, scanning electron microscopy, a hardness tester, an X-ray diffractometer, an electrochemical workstation and an M-2000 frictional wear tester. The results indicated that the nickel-based clad layer without graphene incorporation had the worst forming, with a large number of pores and cracks in the cladding layer. Because graphene agglomerated easily, cracks were regenerated when the content of graphene was higher than 0.5%. The material phases of the cladding layer without graphene incorporation were mainly: Al_3_Ni_2_, Fe_3_Si and SiC. Due to the addition of graphene, the clad layer of specimen 2 was refined and a large number of hard phases, such as CrC and Cr_23_C_6_, were generated, which led to the increase in the hardness of the clad layer. When the content of graphene was further increased, the number of hard phases such as CrC and Cr_23_C_6_ produced in the cladding was relatively reduced due to the agglomeration of graphene, and the hardness of the cladding was reduced. As the impermeability of graphene reduces the diffusion of corrosive media to the substrate, the generation of hard-phase Al_3_Ni_2_ in the cladding layer makes the corrosion resistance of the cladding layer increase with the increase in graphene mass fraction. The result is that, when the content of graphene was 0.5%, the overall performance of the clad layer was the best, where its average hardness was increased by 40%, the average coefficient of friction was reduced by 12.7% and the wear rate was reduced by 60%.

## 1. Introduction

The aviation pressure refueling connector is a key connection component for aircraft refueling and pumping by aircraft refueling trucks. Currently, the material of the aircraft pressure refueling connector and the aircraft refueling port docking area is mainly aluminum alloy. Aluminum alloys have properties such as a high specific stiffness and strength, easy processing and molding [1,2,3]. However, the shortcomings of aluminum alloy, such as its low hardness, poor wear resistance and poor corrosion resistance limit its application under special conditions, such as wear and corrosion [4,5]. In the process of the frequent refueling and docking of aircraft, aviation pressure refueling joints and aircraft refueling joints are prone to damage such as extrusion, scratches and abrasion in the joint area. In addition, aviation pressure refueling joints are also subject to the corrosive effects of aviation oil and airborne media, so the surface of the aircraft refueling interface needs to be protected with surface protection. Traditional aluminum alloy surface strengthening generally takes smelting or surface modification, whereas the aluminum alloy smelting process is more complex and costly. The surface modification technology of aluminum alloy can make aluminum alloy obtain advantages of surface properties that it does not possess, so it becomes a popular research topic in the field of aluminum alloy in recent years.

The existing methods for improving the surface hardness and reducing the friction coefficient of aluminum alloys in aerospace pressure refueling joints are mainly micro-arc oxidation [6,7,8,9], cold gas dynamic spraying [10], electrodeposition technology [11] and laser cladding [12]. The use of micro-arc oxidation can enhance the surface hardness, wear resistance and corrosion resistance of the material. However, the friction coefficient of the micro-arc oxide layer is high and unstable, and the friction reduction effect is not optimal. In addition, it has been found that the coatings prepared by the microarc oxidation method showed a peeling phenomenon [13]. In recent years, the surface strengthening of aluminum alloys by laser cladding is an effective method used to solve its problems, such as a poor wear resistance and easy deformation. Laser cladding technology has the benefits of a high power density, minimal heat-affected zone, dense organization of the clad layer formed by laser cladding and strong bonding between the cladding layer and the substrate [14,15,16,17,18]. Therefore, a wear-resistant and corrosion-resistant cladding layer is prepared at the aircraft refueling interface using laser cladding technology to provide wear reduction, anti-wear and corrosion resistance during operation.

Many researchers have carried out some applied basic research work for laser additive wear-resistant self-lubricating cladding layers and have applied them to parts in the aviation and aerospace industries. However, the development of laser additive wear-resistant self-lubricating cladding applicable to the aluminum alloy surface of aerospace pressure refueling joints is still at the stage of technology accumulation [19,20]. Li et al. [21] prepared Ti/TiBCN coatings on the 7075 aluminum alloy by laser cladding technology. The results showed that the corrosion and wear resistance of the coating was optimal when the TiBCN content was 15 wt%. Xie et al. [22] simulated the temperature and stress fields of the Ni_60_ + Y_2_O_3_ reinforced layer and optimized the process parameters by laser melting experiments. It was found that the combination of finite element numerical simulation to optimize the process parameters of laser melting and rare earth modulation modification could well improve the cracking of the laser-reinforced layer on the surface of aluminum alloy. Wu et al. [23] investigated the organization and wear properties of laser-fused nickel-based composite cladding layers on aluminum alloy surfaces. It was found that the hardness value starts to decrease significantly at the end of the middle of the clad layer. The friction coefficient of the clad layer varies between 0.37 and 0.43 depending on the load, and decreases with an increasing load. Sobiyi et al. [24] made Ti coating on a Ti_6_Al_4_V surface using laser cladding technology. The average speed for all samples was investigated, with the microhardness at the coatings being lower than that of the substrate material. Riquelme et al. [25] prepared an Al/SiC laser cladding layer on AA6082 aluminum alloy. It was found that the composite coating had higher mechanical properties than the substrate. Mattli et al. [26] prepared Al-Inconel625 composites containing varying concentrations of Inconel625 particles. The hardness, compressive strength and tensile strength of the composite coating were significantly increased. In addition, its thermal and dimensional stability was enhanced. Rahman Rashid et al. [27] studied the deposition of 316 L stainless steel cladding of different thicknesses on a mild steel substrate. Significant variations in the cladding thickness, melt pool penetration depth and heat-affected zone depth (HAZ) were observed and correlated with process parameters such as the laser power, scanning speed, dilution and specific energy of the laser beam.

The use of laser cladding technology can improve many properties of aluminum alloys, but, at the same time, there are also some technical difficulties. First of all, because of the high thermal conductivity and low laser absorption properties of aluminum alloys, a higher laser power than other metal substrates needs to be used when melting the cladding layer. Secondly, most of the laser energy is reflected by the aluminum alloy surface during the laser cladding test, which is not conducive to the formation of the molten pool. Again, the coefficient of the thermal expansion of aluminum alloy is relatively high. During the cladding process, the thermal stress at the interface between the aluminum alloy substrate and the cladding layer is high and the bonding force is poor, which will cause the cladding layer to drop off in serious cases. The aluminum alloy contact surfaces of aviation pressure refueling joints require a certain degree of wear resistance. In view of the above technical difficulties, it is necessary to carry out in-depth research on the key technology of laser cladding coating on the aluminum alloy surface.

Although scholars have prepared protective cladding layers on aluminum alloys using a laser, it is a challenge to adjust the corrosion resistance and wear resistance of the cladding layers when preparing them. Silicon carbide has properties such as chemical stability, a high thermal conductivity, a low coefficient of thermal expansion and good wear resistance [28]. Kumar et al. [29] prepared Al7075-2 wt.%SiC micro-nanocomposites using stir friction processing. The improvement was ascribed to the grain size reduction, distribution of SiC nanoparticles uniformly within the matrix, increased particle–matrix interface characteristics and elimination of casting defects such as porosity after the FSP. Jiru et al. [30] investigated the microstructure and microhardness in laser surface alloyed aluminium with TiO_2_ and SiC powders. The ceramic properties of SiC and TiO_2_ improve the microhardness of the alloyed area and achieve a good defect-free surface alloying. Zhang et al. [31] synthesized Ni-SiC composite coatings using ultrasonic electrodeposition. The prepared Ni-SiC composite coatings were found to have a better wear resistance and corrosion resistance than Ni coatings. Huang et al. [32] prepared Ni-SiC composite coatings on A356 aluminum alloy. It was found that the best wear resistance was achieved with a content of SiC particles of 8–10%. Meanwhile, graphene has the advantages of a good electrical conductivity, high strength and good toughness. In addition, graphene can significantly reduce the interfacial adhesion of the coating in sliding contact and lead to a reduction in shear strength [33]. Torres et al. [34] investigated the wear properties of graphene on self-lubricating coatings for high-temperature aluminum forming. Graphene was found to have excellent tribological properties for high-temperature aluminum-forming applications. In addition, although graphene as a lubricating material has a good lubricating effect, it is easy to be oxidized under a high-temperature environment, which makes the self-lubricating effect of the composite coating greatly reduced [35]. Solving the problem of graphene oxidation has been a difficult problem in the industry, and this experiment prevents the oxidation of graphene as much as possible by adding a protective cover during the experiment. In this paper, we investigated the effect of graphene incorporation on the performance of the laser-prepared Ni-based/SiC composite cladding layer to support the further optimization of corrosion and wear resistance at the aircraft refueling interface.

## 2. Materials and Methods

A 6063 aluminum alloy was selected as the substrate material, the dimensions being 50 × 50 × 10 mm; the material composition is shown in Table 1. The substrate material was sanded by 600 # of sandpaper before the test, and then repeatedly cleaned several times with acetone and anhydrous ethanol to remove residual impurities, such as oxides or oil, from the surface of the specimen. Then, it was placed in a dry box to be used. Ni25 powder (99.5% purity, 150–300 mesh), SiC powder (99% purity, 150–300 mesh) and graphene with size of 10–14 μm (99.5% purity) were used as the clad material. Graphene sheet layer material had an aspect ratio of 3:1. The content of graphene was 0 wt%, 0.5 wt%, 1.0 wt% and 1.5 wt%, respectively (see Table 2). The chemical composition of the Ni25 powder is listed in Table 3. Before laser cladding, the raw powders were mixed with a V-18 planetary ball mill for 10 h to ensure homogeneity. The material of the balls used in the ball milling process was ZrO_2_, the diameter was 8 mm and the number was 100.

Through a large number of preliminary experiments, the power of laser should be appropriate. This is because, if the laser power is too low, it will make it difficult for the coating material to fully melt and have good bonding strength with the substrate, and, if the laser power is too high, due to the low melting point of aluminum alloy, it will make the substrate over-melted, thus causing a large dilution rate; in addition, it will also cause the oxidation decomposition of graphene in the coating material. Therefore, the cladding layer was fabricated using a laser cladding system (Nd: LSJG-GQ-6000M3, Ningbo Leisu Laser Technology Co., Ltd., Ningbo, China) with laser power of 2.7 KW, scanning speed of 0.015 m/s, spot diameter of 6 mm, lap rate of 50% and powder feeding rate of 6 g/min, and the cladding layer was prepared using laser on the aluminum alloy substrate. In addition, argon gas with a flow rate of 12 L/min was used to protect the melt pool from oxidation during the laser cladding process, and the powder was injected through a coaxial nozzle coaxially with the laser beam. The distance between the deposition nozzle and the substrate plate was 20 mm, and preparation of single-layer cladding layers used laser cladding. Laser cladding process was conducted using coaxial powder feeding method, and the laser cladding was performed as a single pass process.

After the coaxial laser cladding experiments, metallographic specimens were cut along the vertical direction of the laser scanning direction using a wire cutter (ZhongXin, Taizhou, China) and were polished with 60–2000 mesh SiC sandpaper in that order, and then the metallographic specimens were polished using an MP-2B grinding and polishing machine. Then, the metallographic specimens were etched with aqua regia etching solution for 1 to 3 s. After the experiments, the specimens were characterized by a scanning electron microscope (Phenom g5 pure, Phenom g5 pure, Eindhoven, the Netherlands) with its own energy spectrum, and the compositions of the additive areas were measured by an X-ray diffractometer (Rigaku D/max-2500/PC, Tokyo, Japan), where the operating parameters of the XRD diffractometer were: Cu target, voltage 45 KV, current 200 mA, scanning angle 10–100° and scanning speed 8°/min. The specimen hardness was measured using the HVS-1000Z microhardness tester (LiDun, Shanghai, China) with a load of 500 g and a loading time of 10 s from the surface layer of the cladding layer to the substrate. Finally, the diagonal values of the microscopic traces obtained after unloading were measured and the Vickers hardness values of the material were calculated by the Vickers hardness formula.

The corrosion resistance test of the clad surface in 3.5 wt% NaCl solution was conducted on CHI604E B16323 electrochemical workstation (Huake, Beijing, China), and the test included two parts: potential polarization curve and electrochemical resistance. Electrochemical corrosion experiments cut the substrate and the additive layer size was 10 × 10 mm. Electrochemical corrosion specimens of the working surface, which was sandpaper-polished cladding layer, were polished and cleaned with anhydrous ethanol to remove the residual abrasive and impurities on the surface of the cladding layer, and then cleaned and blow-dried after the use of epoxy resin cold mount for sealing samples. A surface area of 1 cm^2^ of the cladding layer was reserved for electrochemical corrosion test, which was the electrode with Cu wire lead. The reference electrode was saturated glycerol, the auxiliary electrode was platinum sheet and the corrosion resistance of the specimen was characterized by Tafel curve.

The friction and wear test was performed by cutting a specimen of 15 × 15 × 10 mm in length, width and height using wire-cutting machine. In order to have the same roughness of the sample surface, the friction and wear surface was polished with 60–2000 mesh SiC sandpaper in turn, and then the friction and wear test was performed by MPX-3G friction and wear tester (Jinan Hengxu Testing Machine Technology Co., Jinan, China). The experimental parameters are shown in Table 4. The diameter of the ball was 3 mm and the material was silicon carbide ceramic.

## 3. Results and Discussion

### 3.1. The Influence of Graphene Content on Cladding-Layer-Forming Quality

**Figure 1:**
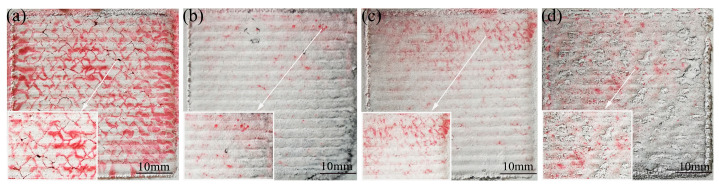
The influence of graphene content on cladding-layer-forming quality: (**a**) 0% graphene mass fraction; (**b**) 0.5% graphene mass fraction; (**c**) 1% graphene mass fraction; (**d**) 1.5% graphene mass fraction.

The influence of the graphene content on the cladding-layer-forming quality is shown in Figure 1. The red highlighted regions on the surface represent pores or cracks exposed by the die penetrant. From Figure 1a, it can be seen that the surface of sample 1 has many areas highlighted in red, which indicates that the surface-forming quality of the cladding layer without the addition of graphene is poor, and that a large number of cracks are produced. As shown in Figure 1b–d, with the increase in the graphene weight fraction, the cracks on the surface of the cladding layer are suppressed. Compared with other specimens, when the weight fraction of graphene is 0.5%, the surface of sample 2 has the least areas highlighted in red, which indicates that it has the least cracks on the surface. For specimen 3, when the weight fraction of graphene reached 1%, the cracks on the surface of the cladding layer started to increase again. This indicates that a certain amount of graphene can suppress the crack generation during laser cladding, but too large a graphene weight fraction will cause the crack to re-emerge. This is due to the uniform distribution of the appropriate amount of graphene in the cladding layer and the formation of carbides precipitating on the grain boundaries, thus acting as a grain refinement, which makes the cladding layer cracks start to decrease. When the weight fraction of graphene is greater than 1%, graphene starts to agglomerate in large quantities, which not only makes its distribution in the cladding layer uneven, but also makes the thermal physical parameters of the composite coating and the substrate differ greatly, and leads to the generation of cracks. The presence of higher amounts of hard phases may lead to an increased brittleness of the clad layer, creating more cracks and flaky fragments in this clad layer [27,36].

### 3.2. Macroscopic Morphology of the Cladding Layer

**Figure 2:**
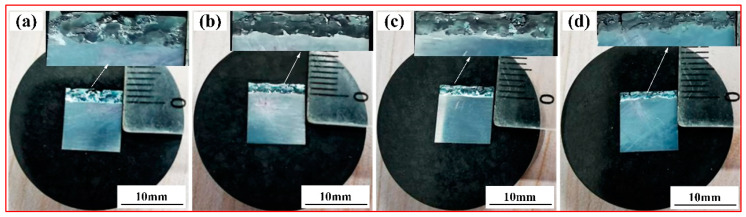
The effect of graphene content on the macroscopic morphology of the cladding layer: (**a**) 0% graphene mass fraction; (**b**) 0.5% graphene mass fraction; (**c**) 1.0% graphene mass fraction; (**d**) 1.5% graphene mass fraction.

The effect of the graphene content on the macroscopic morphology of the cladding layer is illustrated in Figure 2. Figure 2a shows a thickness of the cladding layer of 2 mm for specimen 1 without graphene addition. As observed from Figure 2b–d, with the increase in the graphene weight fraction, the thickness of the cladding layer of specimen 2, specimen 3 and specimen 4 gradually decreased. For specimen 4, when the graphene weight fraction is at 1.5%, the thickness of the cladding layer is at least 1 mm. In addition, the surface of the clad layer without graphene incorporation has many holes, cracks and pores. With the increase in the weight fraction of graphene, especially when the weight fraction of graphene is 0.5%, the holes, cracks and pores on the surface of the cladding layer gradually decrease, and the surface-forming quality is better. Since the laser preparation of the cladding layer uses a coaxial powder feeding method, one part of the energy melts the metal powder and the other part is irradiated directly onto the substrate. The added graphene is a high-melting-point medium: its melting point can be as high as 3652 °C [37]. The addition of graphene high-melting-point media affects the fluidity of the melt pool, and the deposition efficiency of the composite coating gradually decreases with the increase in the graphene content, which makes the thickness of the composite coating start to gradually become smaller. In addition, the high thermal conductivity of graphene makes the melt pool solidify faster, which, in turn, makes the melt pool shallower and leads to a reduction in the thickness of the clad layer. In addition, it can also be seen from Figure 2 that the coating–substrate interface shows an obvious wavy shape as the graphene content increases, whereas the coating without graphene addition shows a flat junction with the substrate, which also indicates that the fluidity of the melt pool decreases with the increase in graphene.

### 3.3. Composition of the Cladding Layer

**Figure 3:**
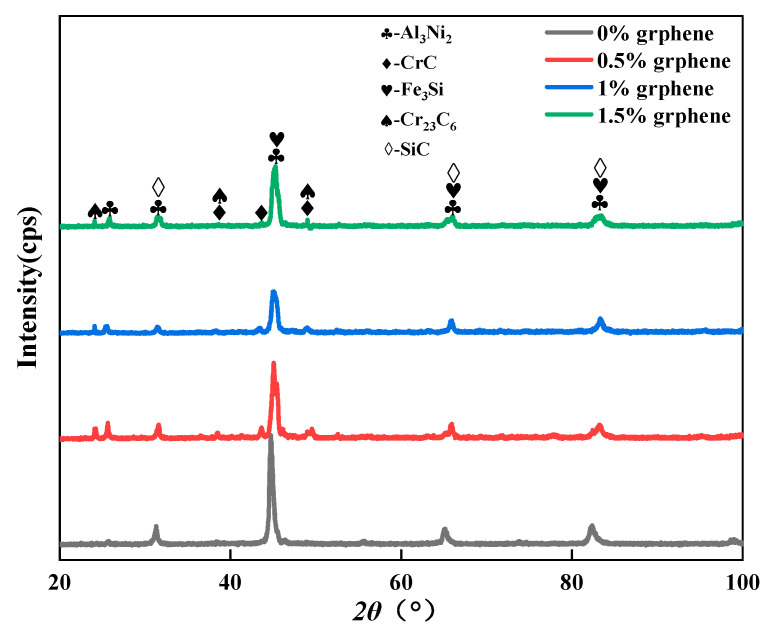
The phase composition of the cladding layer.

Figure 3 shows the XRD patterns of laser cladding. The physical phase composition of the cladding layer was not changed significantly by the increase in graphene. The material phase of the cladding layer mainly consists of intermetallic compounds, such as Al_3_Ni_2_ and Fe_3_Si, and hard phases, such as SiC, CrC, and Cr_23_C_6_. The Al_3_Ni_2_ phase diffraction peak was highest at 2θ = 44°, indicating that the Al_3_Ni_2_ phase grew selectively along this crystal plane, and the growth of the Al_3_Ni_2_ phase along other crystal planes was retarded. Meanwhile, it indicated that diffusion occurred in the melt pool during the cladding process. Due to the low density of aluminum elements, resulting in the aluminum substrate surface microfusion of Al elements into the melt pool inside, Cr and C at high temperatures have a strong tendency to combine, forming CrC, Cr_23_C_6_ and other hard phases. Combined with reference [27], the average lattice parameters of Al_3_Ni_2_ are 4.20922 Å, 5.5361 Å, 5.4429 Å and 4.7266 Å, respectively. The average lattice constants of CrC are 4.3621 Å, 4.9116 Å, 4.7998 Å and 4.4001 Å, respectively. The average lattice constants of Fe_3_Si are 3.4975 Å, 3.8403 Å, 3.4991 Å and 3.6218 Å, respectively. The average lattice constants of Cr_23_C_6_ are 5.7541 Å, 6.8424 Å, 6.5846 Å and 5.8489 Å, respectively. The average lattice constants of SiC are 3.6560 Å, 3.9985 Å, 4.3590 Å and 4.2280 Å, respectively. By comparing the difference, it can be observed that the intensity of the diffraction peaks that was associated with Cr_23_C_6_ varies with the mass fraction of graphene in the fused layer at angles of 2θ = 23.68°, 38.06° and 48.71°. The intensity of the diffraction peak for Cr_23_C_6_ of specimen 1 without graphene addition was almost zero, and the highest intensity of the diffraction peak was observed for Cr_23_C_6_ of specimen 2. Similar trends were observed for the diffraction peaks related to CrC at angles of 2θ = 38.06°, 43.12°and 48.71°, indicating that specimen 2 generated the most CrC and Cr_23_C_6_. Compared to specimen 1, specimen 2 was added to a 0.5% mass fraction of graphene, and the elemental C content was increased in the melt pool; thus the contents of CrC and Cr_23_C_6_ were further increased. Compared to specimens 3 and 4, although the graphene mass fraction was further increased, too high a graphene mass was prone to agglomeration. The agglomerated graphene at high temperatures made it difficult to free the C element to combine with the Cr element in the molten pool; thereby, the contents of CrC and Cr_23_C_6_ were reduced. SiC and Fe_3_Si were detected, indicating that SiC was partially decomposed under the high-temperature irradiation of the laser, and Fe_3_Si was formed by Si and Fe. In the two specimens, there are Al_3_Ni_2_, CrC, Cr_23_C_6_, SiC and other substances that can enhance the corrosion resistance of the surface [38,39].

### 3.4. Microstructure of the Cladding Layer

**Figure 4:**
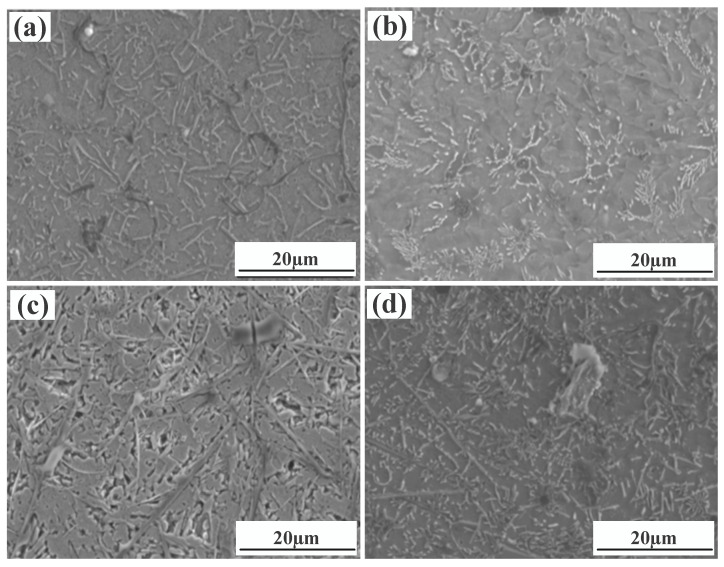
Microstructure of laser cladding Ni25—10%SiC—X% graphene cladding layers: (**a**) specimen 1 (0% graphene content); (**b**) specimen 2 (0.5% graphene content); (**c**) specimen 3 (1% graphene content); (**d**) specimen 4 (1.5% graphene content).

**Figure 5:**
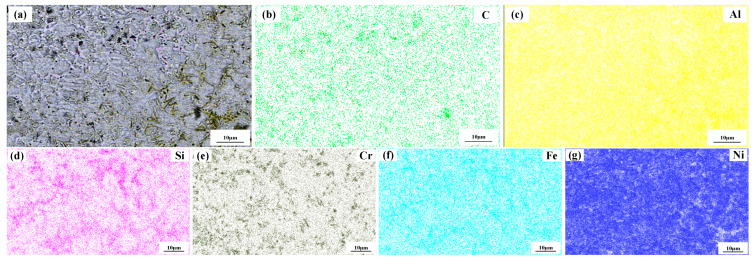
Composition analyses of surface scanning for specimen 2 (0.5% graphene content): (**a**) SEM image; (**b**) C; (**c**) Al; (**d**) Si; (**e**) Cr; (**f**) Fe; (**g**) Ni.

**Figure 6:**
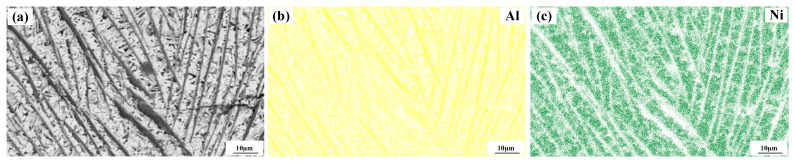
Composition analyses of surface scanning for specimen 3 (1% graphene content): (**a**) SEM image; (**b**) Al; (**c**) Ni.

**Figure 7:**
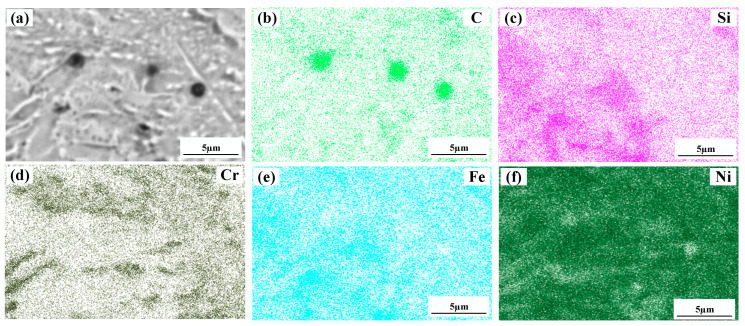
Composition analyses of surface scanning for specimen 3 (1% graphene content): (**a**) SEM image; (**b**) C; (**c**) Si; (**d**) Cr; (**e**) Fe; (**f**) Ni.

**Figure 8:**
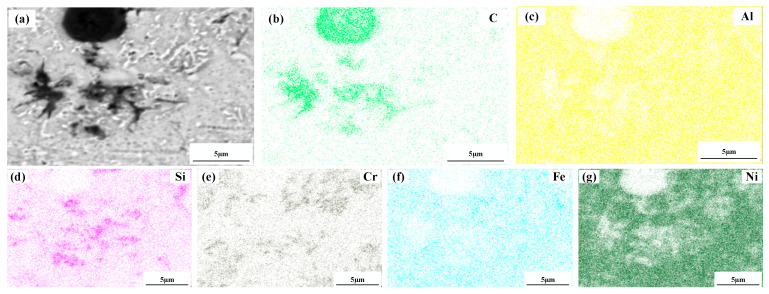
Composition analyses of surface scanning for specimen 4 (1.5% graphene content): (**a**) SEM image; (**b**) C; (**c**) Al; (**d**) Si; (**e**) Cr; (**f**) Fe; (**g**) Ni.

Figure 4 shows a significant difference in the microstructure of the clad layers with different mass fractions of graphene. Regarding specimen 1, where the mass fraction of graphene is 0%, the microstructure of the layer is dominated by irregularly distributed short dendrites. Concerning specimen 2, where the mass fraction of graphene is 0.5%, the grains of the cladding layer are undeveloped petal crystals. In addition, the larger size of the dendritic precipitation phase in the microstructure is reduced, and a fine petal-like microstructure appears. In addition, face scans were performed with an EDS detector. Figure 5 shows the results of the face scan of specimen 2. The distribution of Si, C and Cr elements in the layer is more uniform, which indicates that Si, C and Cr elements are diffused uniformly in the layer. Combined with the XRD of Figure 3, it can be seen that the fused layer of specimen 2 has a large number of hard phases, SiC, CrC and Cr_23_C_6_, that are diffusely distributed. The clad layer is fully strengthened and thus less prone to cracking. This is because the thermal expansion rate of graphene is lower than that of metals, and the addition of graphene can effectively hinder the growth of metal crystals [40]. In addition, the introduction of carbide leads to grain segregation on grain boundaries, which reduces the driving force of Gibb’s free energy [41,42,43]. In turn, the growth of grains is hindered, resulting in a more fine petal-like microstructure and a less dendritic microstructure, which helps to improve the hardness and wear resistance of the clad layer [44]. Regarding specimen 3, where the mass fraction of graphene is 1.0%, the microstructure of the clad layer is dominated by a long dendritic and equiaxed crystal mosaic. Figure 6 shows the results of the face scan of specimen 3. Figure 6 shows that the long dendrite is mainly composed of Al and Ni elements, and the combination of XRD shows that the long dendrite is composed of the intermetallic compound Al_3_Ni_2_. With the increase in the graphene content, the microstructure consisting of Al_3_Ni_2_ in the cladding layer becomes coarse. This is due to the relatively high thermal conductivity of graphene, which can accelerate the solidification rate of the melt pool, thus making the temperature gradient of the whole pool larger. The contribution of graphene thermal conductivity to grain coarseness is greater than the contribution of its low expansion rate to grain fineness. Moreover, the increase in the graphene content caused a partial agglomeration of graphene, which made the contribution of graphene to grain refinement insufficient. The results of the face scan of specimen 3 in Figure 7 show the black circular organization as a large number of aggregates of C elements for graphene agglomeration. In addition, the uneven distribution of Si and Cr elements makes the number of hard phases SiC, CrC and Cr_23_C_6_ generated in the cladding layer fewer, which leads to the weakening of the microstructure-strengthening effect of the cladding layer, and the cladding layer is prone to cracks. Concerning specimen 4, where the mass fraction of graphene is 1.5%, the long dendritic organization in the layer tended to be reduced compared to specimen 3. This is because, in the region of a higher C mass fraction, the nucleation rate of the condensation precipitation phase increases, and the long dendrite organization is relatively fine. Figure 8 shows the greater degree of graphene agglomeration that occurs with a further increase in graphene content. This is due to the larger specific surface area of graphene and the increased volume in contact with each other resulting in greater aggregation. Furthermore, the distribution of Si and Cr elements is similar to that of specimen 3, and the strengthening effect of the clad layer is weakened and thus prone to cracking.

### 3.5. Hardness of Cladding Layer

**Figure 9:**
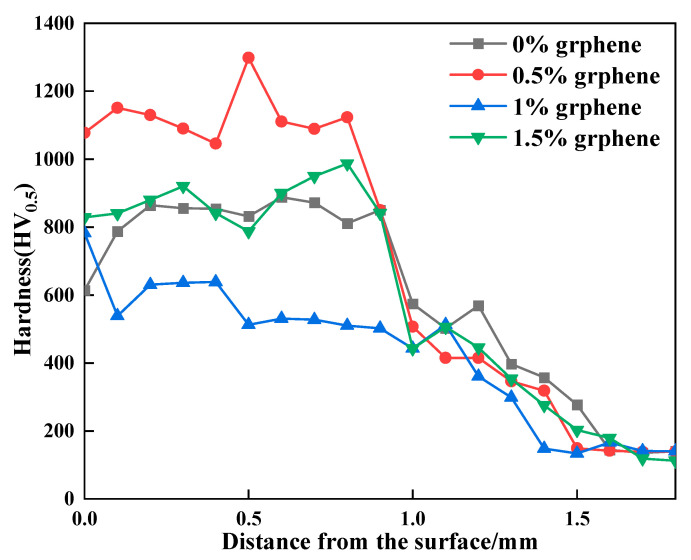
The hardness curves of the clad layer with different additions of graphene.

Figure 9 shows the hardness curves of the clad layer with the different additions of graphene. The hardness values of the clad layer of specimen 1 fluctuate from 574 HV_0.5_ to 902 HV_0.5_. The hardness of specimen 2 fluctuates from 501 HV_0.5_ to 1298 HV_0.5_. The maximum hardness value of 1298 HV_0.5_ occurs in the middle of the clad layer. The hardness of specimen 3 fluctuates from 428 HV_0.5_ to 782 HV_0.5_. The hardness of specimen 4 fluctuates from 592 HV_0.5_ to 884 HV_0.5_. Under the condition of SiC content, the hardness of the clad layer is related to the mass fraction of graphene added. When the addition of graphene is 0.5%, the cladding layer generates a large amount of Al_3_Ni_2_ intermetallic compounds and hard phases such as CrC, Cr_23_C_6_ and SiC. In addition, the combined effect of grain refinement and other factors resulted in the highest hardness of the clad layer of specimen 2, with a maximum value of 1298 HV, which is approximately 6.4 times that of the aluminum alloy base material. When the addition of graphene is 1%, the hardness of the clad layer starts to decrease, and the hardness value is smaller than that of the specimen without graphene addition. This is because the coarse-grained long strips of Al3Ni2 generated in the clad layer of specimen 3 contribute more to the reduction in hardness than the increase in hardness of the small amounts of CrC, Cr_23_C_6_, SiC, etc. generated in the cladding. Thus, specimen 3 exhibits a lower hardness than specimen 1. When the addition of graphene is 1.5%, the long stripes of Al_3_Ni_2_ produced in the clad layer start to become smaller than that of specimen 2. Therefore, the hardness of the clad layer is slightly higher than that of the clad layer, with a graphene mass fraction of 1%.

### 3.6. Corrosion of Cladding Layer

**Figure 10:**
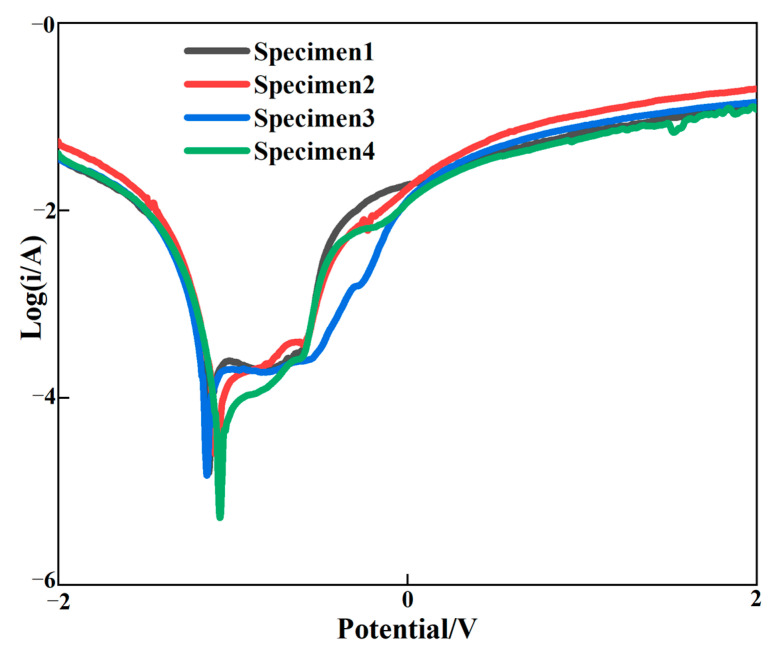
Polarization curve of the cladding layer.

Figure 10 shows the polarization curve of the surface of the cladding layer, and Table 5 shows the electrochemical corrosion performance of the graphene content change the self-corrosion voltage and self-corrosion current density. With the increase in graphene, the self-corrosion potential of the clad layer gradually shifted positively and the self-corrosion current density gradually decreased, which indicated that the corrosion resistance of the clad layer was gradually increased with the increasing mass fraction of graphene. Compared to the cladding layer without graphene incorporation, the positive shift in the self-corrosion potential and the reduction in the current density were more obvious for the cladding layer with graphene addition. This is because the diffusion of corrosive media into the substrate is reduced by the impermeability of graphene. The effective area of the electrochemical interaction between the cladding layer and the corrosive medium was significantly reduced, resulting in the corrosion rate of the cladding layer being decreased and the corrosion resistance of the cladding layer being improved. Secondly, due to the addition of graphene, the pores and crack defects of the cladding layer were improved, the grain was refined and the interface structure was improved. Therefore, corrosion resistance can be improved by strengthening the gain boundaries. Al_3_Ni_2_ in the cladding layer belongs to the corrosion resistance hard phase, which can improve the hardness and corrosion resistance of the cladding layer. Although the molding quality of the cladding layer of specimens 3 and 4 was worse than specimen 2, Al_3_Ni_2_ with corrosion resistance was generated, which improved the corrosion resistance of the cladding layer. As a result, the corrosion resistance of the cladding layer can be significantly enhanced by the addition of graphene.

### 3.7. Wear Properties of the Cladding Layer

**Figure 11:**
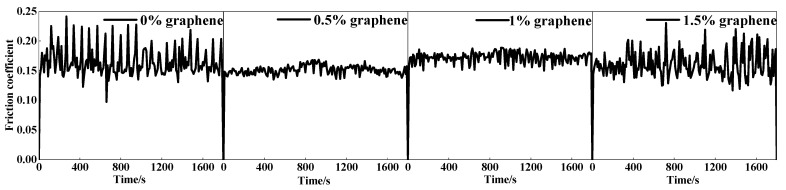
Friction coefficient curve of the cladding layer.

The coefficient of friction (COF) variation curves for the four cladding layers are displayed in Figure 11. The average COF of specimen 1 without graphene addition was 0.682 at the highest, and the COF curve fluctuated widely. The lowest average COF of specimen 2 was 0.598, and the COF curve was relatively smooth. As the graphite mass fraction increases, the COF of the cladding layer starts to increase. When the weight fraction of graphite is 1.5%, the COF of the cladding layer is raised to the maximum, and COF fluctuates considerably. These phenomena are caused by the differing surface morphologies of the cladding layers and by the different graphite contents. Figure 11a shows that specimen 1 has a large number of cracks, which makes the COF higher and fluctuate widely. With the increased mass fraction of graphite, the cracks in the cladding layer are effectively suppressed. In addition, a large amount of graphite was found in the cladding layer. This is because the graphite nodules are decomposed into carbon layers, which are acting as a lubricant during the wear process, which can reduce the COF. For specimen 2, the combination of lubrication from graphite and fewer cracks in the cladding layer resulted in the lowest COF. The number of cracks in the cladding layer increases as the graphite increases in content, which also increases the COF of the cladding layer.

**Figure 12:**
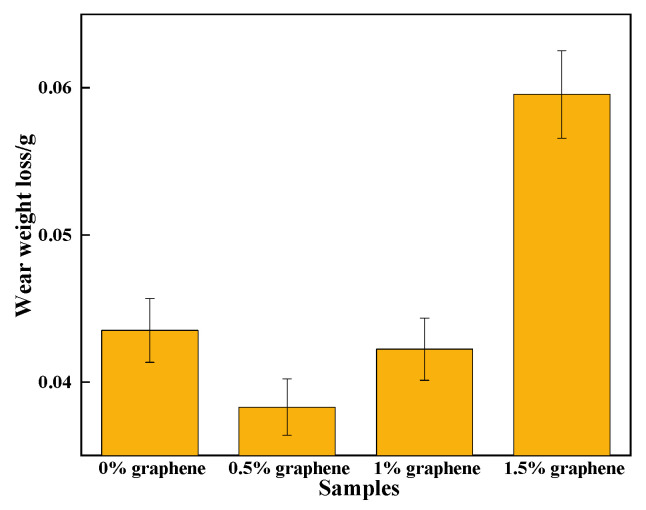
Wear curve of the cladding layer.

Figure 12 displays that the wear amount of specimen 2 is the lowest, and that the wear amount of specimen 1 is 2.5 times that of specimen 2. The hardness of the cladding layer is significantly higher than that of the aluminum alloy material because of the Al_3_Ni_2_ metal compound as the base phase [45]. Due to the addition of the appropriate amount of graphite, many hard phases, including CrC and Cr_23_C_6_ with a high hardness, are generated in the cladding layer. The hard phases can be used as wear-resistant pivot points to reduce the frictional wear of silicon carbide ceramic balls on the cladding layer [46]. They can also play a pegging role in hindering the dislocation movement and yield flow of the cladding layer, and can enhance the plastic deformation resilience of the cladding layer. In turn, it achieves a more effective resistance to the friction and extrusion of silicon carbide ceramic balls. Specimen 2 has the lowest friction coefficient of the cladding layer, with its cladding having the capability to resist plastic deformation during wear, and improves the resistance of the cladding to wear. On the one hand, specimen 2 has a large amount of graphite gathered on the upper surface of the coating, and graphite has a good lubrication effect, which makes the friction coefficient on the surface of specimen 2 lower; on the other hand, specimen 2 has the greatest hardness. Therefore, under the combined influence of a low friction coefficient and high hardness, specimen 2 shows the best wear resistance performance. In addition, the highest wear amount is found in specimen 4 because the wear resistance of the cladding layer is closely related to the COF as well as the hardness. Thus, when the mass fraction of graphite is 1.5%, the COF of the cladding layer is larger and the cladding layer contains some cracks, both of which will increase the wear amount. The above also shows that there is an optimal value of graphene addition. When the content of graphene is low, the hard phase generated in the coating is smaller, thus affecting the hardness of the coating. When the content of graphene is too large, the large amount of the hard phase generated will make the coating more brittle, and it is easy to produce cracks in the surface of the coating; this is similar to the results of Geng’s study [47]. Therefore, in order to obtain the best wear resistance composite coating, the coordination of wear resistance and friction reduction needs to be considered comprehensively.

### 3.8. Wear Mechanism of the Cladding Layer

**Figure 13:**
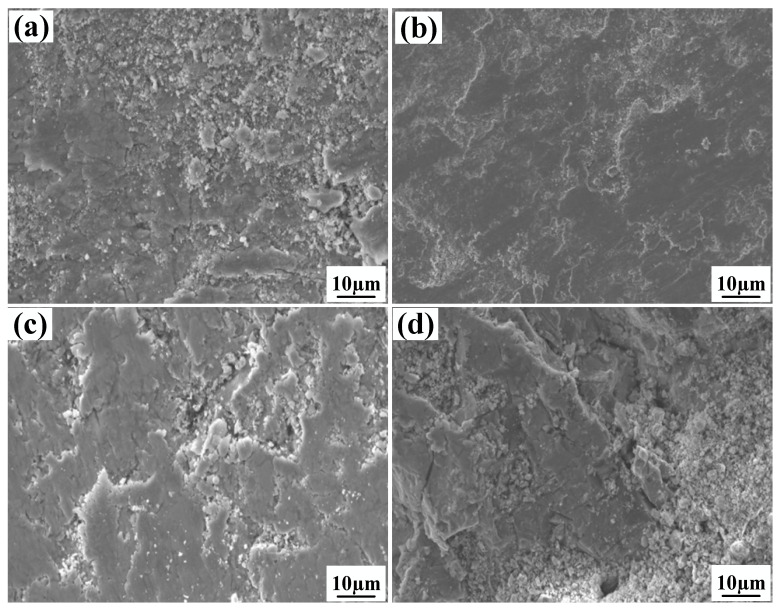
The influence of graphene content on the wear morphology of the coating: (**a**) 0% graphene content; (**b**) 0.5% graphene content; (**c**) 1.0% graphene content; (**d**) 1.5% graphene content.

Figure 13 presents worn morphologies of the clad layer containing different mass fractions of graphene. One can see from Figure 13a that the wear of specimen 1 is typical of multiple plastic transformation wear and adhesive wear. An obvious tissue flaking phenomenon can be observed in the wear, along with the presence of large areas of plastic deformation and powder-like debris, and accompanied by obvious signs of adhesion tearing. This is because, when the graphene mass fraction is 0%, the higher number of SiC ceramic particles leads to an increase in the brittleness of the cladding layer, and cracks are generated in this cladding layer. From Figure 13, it can be seen that the clad layer of specimen 2 is in a slight state of wear, showing slight adhesive wear. There are only a few minor scratches and small abrasions on the wear surface, the tissue flaking on the wear surface is starting to reduce and the wear surface is smoother. This is due to the high hardness and low friction coefficient at a 0.5% graphene mass fraction. The uniform distribution of the hard phase in the clad layer further strengthens the effect of the combined effect of the clad layer. From Figure 13, it can be seen that specimen 3 shows a large tearing phenomenon on the wear surface, which is manifested as severe adhesive wear. The tissue flaking is more serious and accompanied by a small amount of powder-like debris. This is because, when the graphene mass fraction is 1.0%, the coarser Al_3_Ni_2_ intermetallic compound reduces the microhardness of the alloy fused layer, which promotes wear damage. When the mass fraction of graphene is 1.5%, the wear surface of specimen 4 shows a large amount of plastic deformation and heavy tissue flaking. There were a large number of cracks as well as powdered debris on the wear surface; this is due to the large fluctuation in the friction coefficient and the presence of a large number of hard phases (such as SiC, CrC and Cr_23_C_6_) in the fusion cladding layer, which increases its brittleness.

## 4. Conclusions

(1) The phases of the cladding layer without graphene are mainly Al_3_Ni_2_, Fe_3_Si intermetallic compounds and SiC hard phase, and the phases of the cladding layer with graphene are mainly Al_3_Ni_2_, Fe_3_Si compounds and SiC, CrC and Cr_23_C_6_ hard phases. The addition of graphene reduces the crystallinity of the laser cladding layer and refines the cladding layer grains.
(2) When the graphene content is greater than 0.5%, the grain refinement ability decreases. In addition, the presence of excess unmelted graphene weakens the absorption of laser energy in the clad layer, and the hardness and wear resistance of the clad layer are reduced.
(3) When the graphene content was 0.5%, the number of cracks in the cladding layer was the least, and the hardness of the cladding layer was 6.4 times that of the substrate, with a 32.3% increase in hardness, a 27.2% decrease in friction coefficient and a 66.5% decrease in wear rate compared to the cladding layer without graphene addition.

## Figures and Tables

**Table 1:** Powder composition for laser cladding.

Number	Title 2
Specimen 1	90% Ni25 + 10% SiC + 0% graphene
Specimen 2	89.5% Ni25 + 10% SiC + 0.5% graphene
Specimen 3	89% Ni25 + 10% SiC + 1.0% graphene
Specimen 4	88.5% Ni25 + 10% SiC + 1.5% graphene

**Table 2:** Chemical composition of the Ni25 powder (mass fraction, %).

Mn	Cr	O	P	Si	Fe	C	Al	Ni
0.07	3.92	11.95	5.31	3.36	1.14	1.44	0.62	Bal.

**Table 3:** Chemical composition of 6063 aluminum alloy (mass fraction, %).

Mn	Cr	Cu	Mg	Si	Fe	Zn	Ti	Al
0.10	0.10	0.10	0.45–0.9	0.20–0.60	0.35	0.10	0.10	Bal.

**Table 4:** The experimental parameters of wear tests.

Wear Time (min)	Load (N)	Rotation Speed (r/min)	Rotation Radius (mm)	Hardness of Grinding Balls (HV_0.5_)
30	30	300	1.5	1400

**Table 5:** Self-corrosion voltage and self-corrosion current density of the cladding layer.

Number	Self-Etching Voltage Ecorr/V	Self-Etching Current Density 10^−4^ A·cm^−2^
Specimen 1 (0% graphene)	−1.138	2.537
Specimen 2 (0.5% graphene)	−1.101	2.246
Specimen 3 (1% graphene)	−1.092	2.164
Specimen 4 (1.5% graphene)	−1.084	2.152

## Data Availability

Not applicable.
